# HIV and inflammatory markers are associated with persistent COVID‐19 symptoms

**DOI:** 10.1002/iid3.859

**Published:** 2023-05-08

**Authors:** Patrick Kamanzi, Gina Mulundu, Keagan Mutale, Chibamba Mumba, Owen Ngalamika

**Affiliations:** ^1^ Dermatology and Venereology Division, Department of Medicine, University of Teaching Hospital University of Zambia School of Medicine Lusaka Zambia; ^2^ Department of Pathology and Microbiology, University of Teaching Hospital University of Zambia School of Medicine Lusaka Zambia; ^3^ HHV‐8 Molecular Virology Laboratory University Teaching Hospital Lusaka Zambia

**Keywords:** biomarker, HIV, immunological factors, long COVID

## Abstract

**Background:**

A proportion of COVID19 survivors may present with long‐COVID, which is persistent symptoms lasting four or more weeks post SARS‐CoV‐2 infection. These symptoms may be mild to severe, and may affect different organ‐systems of the body.

**Aims:**

The main objective of this study was to determine the demographic, clinical and immunological factors associated with long COVID.

**Materials & Methods:**

We conducted a nested case control study, with a total of 94 study participants initially included, and 64 participants matched for age and sex for biomarker analyses.

**Results:**

32/94 (34.1%) of all the participants had long COVID. Respiratory symptoms were the most common (59.5%) followed by the musculoskeletal symptoms (28.1%). HIV was an independent predictor of long COVID (adjusted odds ratio = 2.7; *p* = .037). In all the 64 matched cases and controls, IFN‐β was significantly higher among controls than cases. After stratifying by HIV, IL6 was significantly higher among cases than controls in the HIV‐ group (2.06 vs. 0.81 pg/mL; *p* = .02). On the other hand, IFN‐β was significantly higher among controls than cases in the HIV+ group (251 vs. 0 pg/mL; *p* = .01).

**Conclusion:**

HIV infection is a risk factor for long COVID, and inflammatory markers associated with long COVID may be slightly different for HIV− and HIV+ individuals.

## Graphical abstract

A proportion of coronavirus disease 2019 (COVID‐19) survivors may present with long‐COVID, which is persistent symptoms lasting four or more weeks post severe acute respiratory syndrome coronavirus 2 infection. The main objective of this study was to determine the demographic, clinical, and immunological factors associated with long COVID. Human immunodeficiency virus (HIV) infection is a risk factor for long COVID, and inflammatory markers including interleukin(IL)‐6 and interferon (IFN)‐β are associated with long COVID.

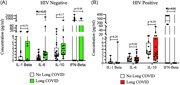

## 1 INTRODUCTION

Coronavirus disease 2019 (COVID‐19) is the symptomatic manifestation of severe acute respiratory syndrome coronavirus 2 (SARS‐CoV‐2) virus infection. SARS‐CoV‐2 is an enveloped RNA β coronavirus that infects host cells via the angiotensin‐converting enzyme 2 receptor.^1^ As of December 2020, a global estimate of over 600 million cases and over 6.5 million deaths have been attributed to COVID‐19.^2^, ^3^ Upon achieving herd immunity from infections and vaccinations, most countries worldwide have experienced a significant drop in SARS‐CoV‐2 infections and COVID‐19‐related deaths. However, some survivors of COVID‐19 are experiencing persistent symptoms that include but are not limited to fatigue, difficulties in breathing, chest pain, joint pain, headache, and sleep disturbance.^4^

The persistent symptoms of COVID‐19 have several definitions according to different international organizations. One of the most commonly used and has been adopted for this study is the US Center for Disease Control (CDC) definition.^5^ CDC refers to the persistent symptoms as post‐COVID or long‐COVID and defines them as symptoms lasting four or more weeks post‐SARS‐CoV‐2 infection.

Acute COVID‐19 and its pathophysiology are well documented. However, there is a paucity of knowledge and information regarding long COVID and its pathophysiology. Currently, it is hypothesized that long COVID is driven by a combination of factors including viral persistence, autoimmunity, persistent aberrant inflammation coupled with organ damage during acute COVID‐19, and exacerbation of underlying medical and psychiatric illnesses.^6^ Infection with human immunodeficiency virus (HIV) is a medical condition that may increase the risk of long COVID.^7^ People living with HIV (PLWH) are at risk of developing various chronic comorbid conditions including postviral syndromes due to their immune dysregulation. HIV infection leads to an immune activation resulting in a proinflammatory state, that may persist even with antiretroviral therapy treatment.^8^ This abnormal immunological state in PLWH may increase their risk of developing long COVID. We hypothesize that proinflammatory markers are elevated while anti‐inflammatory markers are lower in individuals with long COVID.

Since long COVID has had several different manifestations involving multiple organ systems of the body with poorly defined pathobiological mechanisms, recommendations regarding laboratory testing and specific management are currently lacking. The aim of this study was to identify the biomarkers associated with long COVID and how HIV and other comorbidities may predispose individuals to develop long COVID. We investigated and compared pro‐ and anti‐inflammatory cytokine levels between individuals with and without long COVID. To the best of our knowledge, there were no published studies on inflammatory markers and long COVID in HIV at the time this study was conducted.

## 2 MATERIALS AND METHODS

### 2.1 Study design and population

This was a nested case‐control study that recruited participants who previously suffered from COVID‐19 and were part of a prospective cohort study conducted at the Adult Hospital of the University Teaching Hospital in Lusaka, Zambia. The participants in the parent study had a reverse transcriptase‐polymerase chain reaction‐positive SARS‐CoV‐2 test.^9^ They were adults aged 18 years old and above. For this current study, they were selected at the 8‐week follow‐up time‐point, approximately 18 weeks from the time they tested positive for SARS‐CoV‐2. The selected participants had no symptoms of long COVID before testing positive for SARS‐CoV‐2 and before enrollment in the parent study. Our definition of long COVID was according to the CDC definition of signs, symptoms, and conditions that are present 4 weeks or more after the initial phase of infection. However, our study population also met the World Health Organization criteria of symptoms lasting 3 or more months after the initial infection. Clinical and sociodemographic information as well as venous whole blood from the main study was used. Among the symptoms defined as long COVID were respiratory (cough, dyspnea, chest pain), cardiovascular (easy fatigability, heart palpitations, chest tightness), neurological (dizziness, headache, sleep disturbance, loss of taste and/or smell), and musculoskeletal (joint pains, muscle pain).

Ninety‐four participants were found to have been successfully followed up in the main study. To investigate and compare the inflammatory biomarkers, 64 (31 with long COVID and 33 without long COVID) were matched for sex and age at a ratio of approximately 1:1. All the biomarkers were assessed at the time of follow‐up in the main study at approximately 4.5 months from time of SARS‐CoV‐2 infection. This study was approved by the University of Zambia Biomedical Research Ethics Committee (Reference Number 3412‐2022) and the National Health Research Authority of Zambia.

### 2.2 CD4 counting and HIV viral loads

CD4 counts and HIV viral loads had been done previously at the time of follow‐up in the parent study cohort. Quantification of CD4 counts and HIV viral loads were done using the BD TriTest kit (BD biosciences) on a 4‐color BD FACSCalibur (BD Biosciences) and the Aptima HIV‐1 Quant Dx Assay Kit on a Hologic Panther (Hologic), respectively, as specified by the manufacturer's protocol.

### 2.3 Cytokine assays

Plasma samples stored at −80°C, obtained from the parent study were used for the cytokine assays. The MILLIPLEX MAP human cytokine magnetic bead panels (IL‐1β, IL‐ 6, and IL‐10) and (IFN‐β) were used to perform the assays as per the manufacturer's instructions. For IFN‐β, a fourfold dilution of plasma samples was done as per the manufacturer's instructions. Samples were run in duplicate, with the mean value obtained for final analysis. Data acquisition was performed on a Luminex MAGPIX instrument (Luminex Corporation) with xPONENT version 4.3 software. The cytokines were selected based on previous studies that suggested that they were associated with long COVID status.^10^

### 2.4 Statistical analysis

STATA version 17 statistical software was used for analysis. Baseline characteristics were analyzed using descriptive statistics. The distribution of variables in groups was expressed as a median and interquartile range while categorical or binary variables were as percentages. The chi‐square test was used to determine the association between long COVID and dichotomous predictors. The Mann–Whitney test was used to determine any association between continuous variables and the long COVID status. Multivariate logistic regression was used to control for potential confounders. Parameters used for inclusion of variables in the multivariate logistic regression model and selection of the best model included the *p* values of 0.3 or below on bivariate analysis, the area under the curve with or without the variables in the model, and the Akaike's and Bayesian information criteria. Variables with *p* values .3 or below were included because they improved the model with regard to the pseudo‐*R* ^2^ and standard error. A Hosmer–Lameshow goodness of fit test was done to determine whether the data fit the model. *p* < .05 were considered statistically significant.

## 3 RESULTS

### 3.1 Characteristics of study participants

Ninety‐four (94) study participants who were successfully followed up in the parent study were included in this study. Sixty‐two (65.9%) of these participants had no long COVID while 32 (34.1%) had long COVID symptoms. The baseline characteristics including demographics, clinical, and HIV status were not significantly different between the two groups (Table 1). However, the group without long COVID had a marginally significant higher proportion of fully vaccinated individuals compared to the group with long COVID (69.4% vs. 50%; *p* = .07). For the analysis of inflammatory markers, the 31 individuals with long COVID were matched with 33 individuals without long COVID by age and gender. The baseline characteristics between these two matched groups were not significantly different (Supporting Information: Table 1).

**Table 1:** Baseline characteristics of study participants by long COVID‐19 status.

	No long COVID (*N* = 62)	Long COVID (*N* = 32)	*p* Value
Age (years)	49 (42–57)	52 (45.5–59)	.32
Males	41.9%	34.4%	.51
BMI (kg/m^2^)	29.1 (24.7–33.1)	26.2 (25.7–29.4)	.45
HIV positive^a^	20/57 (35.1%)	56.3%	.05
HIV viral load (copies/mL)	0 (0–0)	0 (0–0)	.64
CD4 count (cells/µL)	622 (346–827)	492 (181–699)	.17
Hypertensive	41.9%	31.3%	.31
Diabetic	8.1%	0%	.10
Hospitalized for COVID‐19	58.1%	46.9%	.30
Fully vaccinated for COVID‐19	69.4%	50%	.07

*Note*: CD4 counts and HIV viral loads are only compared between the HIV+ individuals in the two groups.

Abbreviations: COVID‐19, coronavirus disease 2019; HIV, human immunodeficiency virus.

^a^ Excludes five individuals with diabetes in the long COVID group.

### 3.2 Manifestations of long COVID

**Figure 1:**
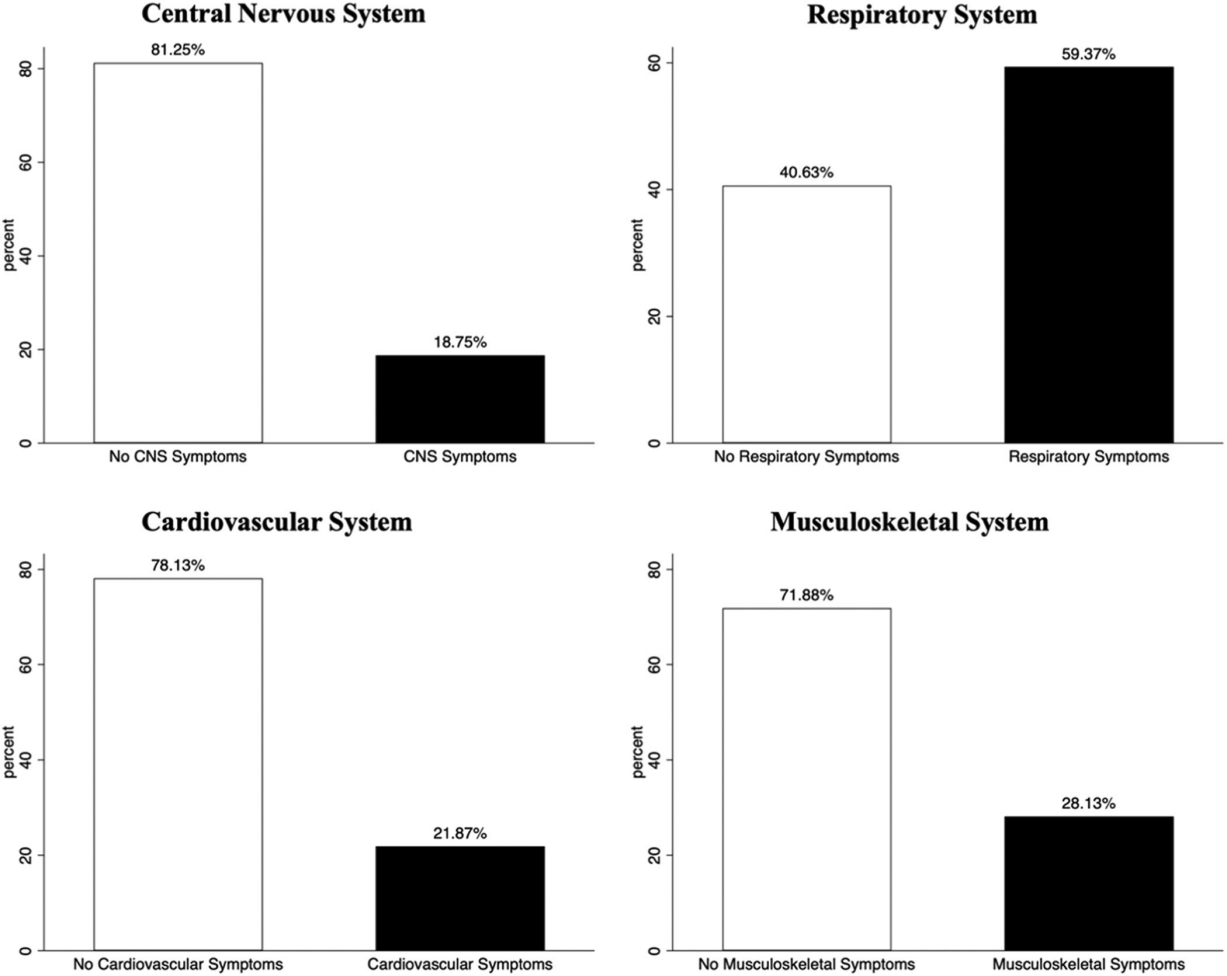
Common organ systems manifesting as long COVID symptoms. The respiratory system is the most commonly affected followed by the musculoskeletal system. COVID, coronavirus disease 2019.

Among the manifestations of long COVID, there were multiple organ systems involved. Persistent symptoms involving the respiratory system were the most common, followed by musculoskeletal, then cardiovascular, then the central nervous system (Figure 1). Respiratory manifestations included cough, chest pain, and difficulties in breathing. Cardiovascular manifestations included easy fatigability and chest tightness. Neurological symptoms included dizziness, headache, sleep disturbance, and loss of taste and smell. The most prominent musculoskeletal symptoms were joint and muscle pains. Eight (25%) of participants with long COVID had more than one organ‐system affected.

### 3.3 HIV independently associated with long COVID

None of the baseline demographic and clinical characteristics were associated with long COVID on bivariate analysis. After adjusting for vaccination status and previous COVID‐19 hospitalization while excluding the five diabetics in the study, HIV was independently associated with about 2.7 higher odds of long COVID (adjusted odds ratio = 2.72 [1.1–6.9]; *p* = .037). Diabetes was excluded as there were few diabetics in the study and most of them (4/5) were HIV positive which highly confounded the analysis of the HIV association in both univariate and multivariate analysis.

### 3.4 Inflammatory markers associated with long COVID

We compared the levels of the pro‐ and anti‐inflammatory cytokines including IL‐1β, IL‐6, IL‐10, and IFN‐β between individuals with and without long COVID. We observed that levels of IL‐1β, IL‐6, and IL‐10 were not significantly different between the two groups (Table 2). However, IFN‐β was significantly higher among individuals without long COVID symptoms compared to those with long COVID manifestations.

**Table 2:** Comparisons of inflammatory markers between cases and controls.

	No long COVID (*N* = 33)	Long COVID (*N* = 31)	*p* Value
IL‐1β (pg/mL)	0 (0–0.53)	0 (0–0.26)	.73
IL‐6 (pg/mL)	0.91 (0.47–1.56)	1.48 (0.64–2.76)	.17
IL‐10 (pg/mL)	2.63 (1.50–4.83)	2.59 (1.81–7.10)	.65
IFN‐β (pg/mL)	0 (0–371)	0 (0–0)	**.017**

*Note*: Bold value indicates statistically significant.

Abbreviations: COVID, coronavirus disease; IFN, interferon; IL‐6, interleukin 6.

**Figure 2:**
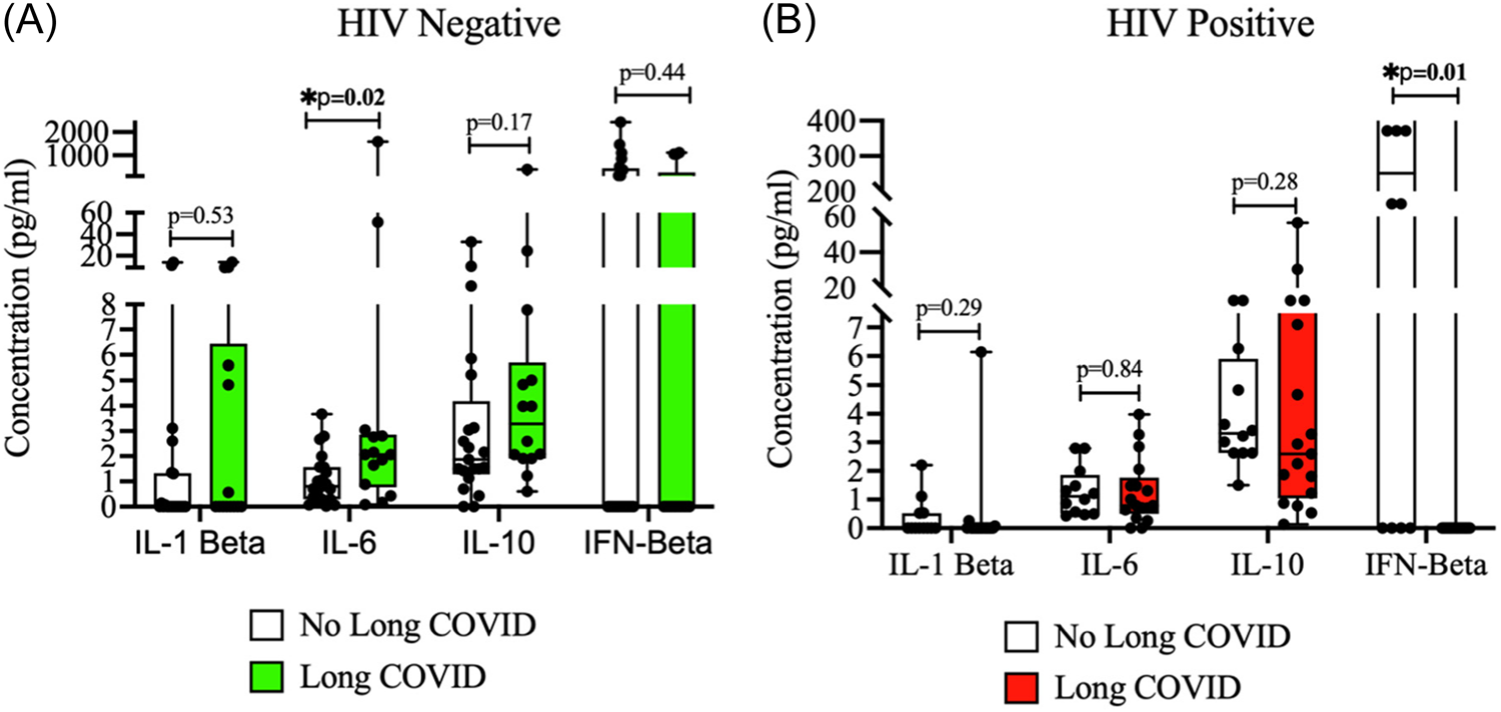
Inflammatory markers associated with long COVID by HIV status. (A) Plasma IL‐6 levels were significantly higher among cases than controls in the HIV− group. (B) Plasma IFN‐β levels were significantly higher in the controls than in cases in the HIV+ group. COVID, coronavirus disease; HIV, human immunodeficiency virus; IFN, interferon; IL‐6, interleukin 6.

Since HIV was an independent predictor of long COVID, we conducted a subanalysis to compare the inflammatory markers associated with long COVID by HIV status. We observed that within the HIV− group, IL‐6 was significantly higher in the individuals with long COVID compared to the group without long COVID symptoms (Figure 2 and Supporting Information: Table 2). In the HIV+ group, IFN‐β was significantly higher in individuals without long COVID symptoms compared to those with long COVID. The other markers were not significantly different between the cases and controls in the two subgroups.

## 4 DISCUSSION

The purpose of this study was to identify demographic, clinical, and inflammatory markers associated with long COVID. We observed that among the clinical and demographic factors, HIV was an independent predictor of long COVID and associated with 2.7 times higher odds of long COVID. A recent study by Pujari et al. observed and reported that long COVID was very common among PLWH.^7^ In another study, it was observed that a positive HIV status was associated with four times higher odds of having long COVID.^11^ It appears from this study and others that there are factors associated with HIV infection such as chronic immune activation and immune dysregulation that could increase the risk of long COVID. This is because HIV on its own has been previously associated with such chronic symptoms even in the pre‐COVID era in our population.^12^

The observed long COVID symptoms were many and involved several organ systems. The most common were respiratory followed by musculoskeletal symptoms, then cardiovascular and central nervous systems. In one large cohort study, respiratory and neurological symptoms were among the most commonly observed manifestations of long COVID.^13^ Other studies have also reported that respiratory, musculoskeletal, cardiovascular, and neuropsychiatric symptoms are among the most common long COVID manifestations in different settings.^14^, ^15^ It is not surprising that respiratory symptoms are often the most common long COVID manifestations as SARS‐CoV‐2 is an infection of the respiratory system and recovery of the affected tissues may result in long‐term sequelae or complications such as fibrosis of lung tissue, reduced lung volume, and persistent interstitial lung disease.^16^, ^17^, ^18^

When pro‐ and inflammatory markers were compared between all cases with long COVID and controls without long COVID, it was observed that IFN‐β was higher in the controls than in the cases (*p* = .017). A subanalysis by HIV status indicated that high plasma IL‐6 levels were associated with long COVID among HIV− individuals, while plasma IFN‐β levels were significantly higher among HIV+ individuals without long COVID. IL‐6 is a pleiotropic cytokine with both pro‐ and anti‐inflammatory effects. However, in the case of long COVID, it appears to have proinflammatory effects, and it has also been observed by other studies to be persistently elevated in the plasma of long COVID patients.^19^ IL‐6 is known to increase T helper 17 activity and hence reduce regulatory T cell activity, and this imbalance is associated with a systemic inflammatory state that can result in long COVID symptoms.^20^, ^21^ IFN‐β is a type 1 interferon that has both antiviral and immunoregulatory functions, including both pro‐ and anti‐inflammatory effects.^22^, ^23^ The mechanisms for the anti‐inflammatory effects of IFN‐β include the downregulation of class 2 human leukocyte antigen, inhibition of T cell migration, and upregulation of IL10 secretion.^24^

Our observation that high IFN‐β is protective from long COVID in HIV+ individuals, while high IL‐6 is a maker of long COVID in HIV− individuals requires further studies with larger sample sizes. This is especially required for the study of IFN‐β in the HIV− population because it is significantly raised in the controls for the entire study population, and is detectable in the controls and not cases for the HIV− population. Nevertheless, HIV+ individuals may be in a proinflammatory immune‐dysregulated state due to the chronic immune activation associated with HIV infection that may raise proinflammatory markers, and this state of inflammaging may make it difficult to distinguish certain markers such as IL‐6 between cases and controls of the HIV+ population.^25^, ^26^ Therefore, inflammatory biomarkers and potential therapeutic targets for long COVID may be slightly different in HIV− and HIV+ individuals. We did not observe any significant difference in the other markers (IL‐1β and IL‐10), possibly due to the small sample size.

## 5 STUDY LIMITATIONS

Some of the limitations of this study included the unavailability of some information as it was a nested study that used samples and information previously collected. For instance, we were not able to obtain some other relevant information such as the smoking status of the patients in the database. Also, the sample size was not large enough to perform some other sub‐analyses. In addition, we were unable to assess potential persistent viral shedding.

## 6 CONCLUSION

Long COVID is common among individuals recovering from acute SARS‐CoV‐2 infection. There are many long COVID symptoms associated with different organ systems, among which symptoms of the respiratory system are the most common. HIV infection is independently associated with a high risk of long COVID. High IL‐6 is associated with long COVID in the HIV− population while high IFN‐β is protective from long COVID in the HIV+ population.

## AUTHOR CONTRIBUTIONS

Patrick Kamanzi was involved in the conceptualization of the study, and also conducted the experiments, and drafted the manuscript. Keagan Mutale was involved in conducting the experiments, and reviewed and edited the manuscript. Gina Mulundu was involved in conceptualizing the study idea, and also reviewed and edited the manuscript. Chibamba Mumba was involved in conceptualizing the study and also reviewed and edited the manuscript. Owen Ngalamika was involved in the conceptualization of the idea, designing the experiments, supervising the work, data analysis, and reviewing and editing the manuscript. All authors have read and agreed to the published version of the manuscript.

## CONFLICT OF INTEREST STATEMENT

The authors declare no conflict of interest.

## ETHICS STATEMENT

The use of data and samples for this study was approved by the University of Zambia Biomedical Research Ethics Committee (REF. NO. 3412‐2022) and the Zambia National Health Research Authority.

## Supporting information

Supporting information.

## Data Availability

Anonymized data can be made available upon reasonable request to the corresponding author.
