# Supplementary material for: HIV and inflammatory markers are associated with persistent COVID‐19 symptoms
Source: Immun Inflamm Dis. 2023 May 8;11(5):e859. doi: 10.1002/iid3.859 (PMC10165949; doi:10.1002/iid3.859)
Supplement: Supplementary file 1 — Supporting information. [file IID3-11-e859-s001.docx]

| **Table S1**. Baseline characteristics of matched participants by long COVID status | | | |
| --- | --- | --- | --- |
|  | **No Long Covid**  **(N=33)** | **Long Covid**  **(N=31)** | **p Value** |
| Age (Years) | 50[45-58] | 52[44-59] | 0.72 |
| Males | 36.4% | 35.5% | 0.94 |
| BMI (kg/m^2^) | 29[24.3-32.1] | 26.5[25.5-30.4] | 0.63 |
| HIV Positive | 36.4% | 54.8% | 0.14 |
| HIV Viral Load (copies/ml) | 0[0-0] | 0[0-0] | 0.33 |
| CD4 Count (cells/µl) | 512[316-865] | 544[306-699] | 0.13 |
| Hypertensive | 51.5% | 29% | 0.07 |
| Diabetic | 12.1% | 0% | 0.05 |
| Hospitalized for COVID19 | 57.6% | 45.2% | 0.32 |
| Fully Vaccinated for COVID19 | 66.7% | 51.6% | 0.22 |
| CD4 counts and HIV viral loads are only compared between the HIV+ individuals in the two groups. | | | |

| **Table S2**. Comparisons of Inflammatory markers between cases and controls by HIV status | | | |
| --- | --- | --- | --- |
| **HIV-** | | | |
|  | **No Long Covid**  **(N=21)** | **Long Covid**  **(N=14)** | **p Value** |
| IL-1β (pg/ml) | 0[0-1.31] | 0[0-5.59] | 0.53 |
| IL-6 (pg/ml) | 0.81[0.29-1.56] | 2.06[0.88-2.80] | **0.023** |
| IL-10 (pg/ml) | 1.87[1.42-3.13] | 3.29[1.91-5.01] | 0.17 |
| IFN-β (pg/ml) | 0[0-371] | 0[0-0] | 0.44 |
| **HIV+** | | | |
|  | **No Long Covid**  **(N=12)** | **Long Covid**  **(N=17)** | **p Value** |
| IL-1β (pg/ml) | 0[0-0.52] | 0[0-0] | 0.29 |
| IL-6 (pg/ml) | 1.11[0.53-1.73] | 0.79[0.64-1.48] | 0.84 |
| IL-10 (pg/ml) | 3.32[2.63-5.55] | 2.59[1.22-7.10] | 0.28 |
| IFN-β (pg/ml) | 251[0-436] | 0[0-0] | **0.006** |
